# Characterising the cavitation activity generated by an ultrasonic horn at varying tip-vibration amplitudes

**DOI:** 10.1016/j.ultsonch.2020.105273

**Published:** 2020-08-06

**Authors:** Lukman Yusuf, Mark D. Symes, Paul Prentice

**Affiliations:** a Cavitation Laboratory, Centre for Medical and Industrial Ultrasonics, University of Glasgow, Glasgow G12 8QQ, United Kingdom; b WestCHEM, School of Chemistry, University of Glasgow, University Avenue, Glasgow G12 8QQ, United Kingdom

**Keywords:** Ultrasonic horn, Cavitation, Shock wave, Subharmonic, Vibration amplitude

## Abstract

Dual-perspective high-speed imaging and acoustic detection is used to characterise cavitation activity at the tip of a commercial 20 kHz (*f_0_*) ultrasonic horn, over 2 s sonications across the range of input powers available (20 – 100%). Imaging at 1 × 10^5^ frames per second (fps) captures cavitation-bubble cluster oscillation at the horn-tip for the duration of the sonication. Shadowgraphic imaging at 2 Mfps, from an orthogonal perspective, probes cluster collapse and shock wave generation at higher temporal resolution, facilitating direct correlation of features within the acoustic emission data generated by the bubble activity. *f_0_/m* subharmonic collapses of the primary cavitation cluster directly beneath the tip, with *m* increasing through integer values at increasing input powers, are studied. Shock waves generated by periodic primary cluster collapses dominate the non-linear emissions of the cavitation noise spectra. Transitional input powers for which the value of *m* is indistinct, are identified. Overall shock wave content within the emission signals collected during sonications at transitional input powers are reduced, relative to input powers with distinct *m*. The findings are relevant for the optimisation of applications such as sonochemistry, known to be mediated by bubble collapse phenomena.

## Highlights

• Cavitation is interrogated via dual-perspective high-speed imaging and acoustic detection.
• Input power is sampled in fine increments.
• Primary cluster collapses occur subharmonically.
• The integer order of subharmonic response, *m*, generally increases with increasing power.
• Time-averaged shock wave emission is reduced at transitional powers for which *m* is indistinct.

## 1 Introduction

The ultrasonic horn, also known as the sonotrode, is one of the most common laboratory-based acoustic devices, second only perhaps to the cleaning bath. During operation, mechanical vibrations from a piezoelectric element or element stack, within the main housing of the device, generally at a fundamental driving frequency (*f_0_*) between 20 and 30 kHz, are transmitted and amplified through a tapered or stepped metallic rod. The (usually planar) tip of the rod therefore vibrates with a displacement amplitude in the range of several microns to several 100′s µm dependent on the input power setting, at *f_0_*.

Horn-type devices with a range of powers and tip diameters have been reported as being utilised for processes such as cell disruption or lysis [1], [2], preparation and dispersion of emulsions [3] and nanoparticles [4], and the precipitation of ‘sonocrystallisation’ [5], for applications ranging from pharmaceutical and food sciences, to biodiesel production [6] and water treatment [7], [8]. Applications based in the medical sector, at various stages of development, include enhanced tissue dissection for surgery [9], transdermal drug delivery [10] and the generation of (ultrasound contrast agent) microbubble suspensions [11].

In applications during which the tip is immersed in liquid, it is generally accepted that cavitation in the region of the tip makes a significant contribution to the desired effect of the sonication [12]. Intuitively, frequency of operation, tip-size and input power, which determines the amplitude of tip-vibration, may be expected to have significant influence on the characteristics and behaviour of the cavitation generated. Other factors known to affect bubble activity include the properties of the liquid host medium, such as dissolved gas content and viscosity, [13].

Accordingly, there has been significant interest in characterising the cavitation activity generated by horns, both for identifying process mechanisms and optimising applications [14]. As reviewed below, direct optical imaging and acoustic detection of the emission signal generated by the cavitation activity have underpinned much of this research effort.

Moussatov *et al.* [15] identified a cone-like bubble structure at the tip of a 20.7 kHz horn for three different tip diameters (20, 80 and 120 mm-Ø). The cone structure is thought to generate via self-focusing through a bubble-layer at the surface, thickest at the centre [16]. The former report also confirmed strong sonochemical activity within the cone, via chemiluminescence observation in luminol, in agreeance with an earlier study [17], investigating chemiluminescence around tips of different shapes.

Other studies have sought to further characterise the dynamic cavitation activity at the vibrating tip, based on high-frame rate imaging, often in conjunction with hydrophone measurements of the acoustic emissions. Birkin *et al.* [18] studied the primary cavitation cluster, beneath a 3 mm-Ø tip at *f_0_* = 23 kHz, and at a single intensity of ~ 50 Wcm^−2^ (measured via the calorific technique, but determined by the tip-vibration amplitude). A principle finding was that the bubble cluster underwent periodic collapses at subharmonic frequencies (*f_0_/3* and *f_0_/4*), which corresponded to the detection of pressure spikes in the acoustic measurements. It was further shown that surface erosion effects (via a passivated electrode located close to the tip) and sonoluminiescence also correlated temporally to the cluster collapses, pointing to the importance of the bubble collapse in cavitation-mediated processes. Žnidarčič *et al.* [19] made equivalent observations of the cavitation bubble activity around a 3.2 mm-Ø tip, at tip-vibration amplitudes of 100, 132 and 164 μm. The authors distinguished the large vaporous bubble activity they observed, attached to and covering the tip as ‘acoustic supercavitation’. Nonetheless, pressure spikes at *f_0_/3* and *f_0_/4* were reported for the lowest and highest tip amplitudes, respectively, corresponding to subharmonic oscillations of the bubble activity. At an intermediate value for tip vibration, it was noted that the cavity oscillation period shifted irregularly between 3 and 4 cycles of the tip-vibration, or that cavity oscillation frequency was not clear. These studies provide insights into the cavitation dynamics at the tip, but full characterisation was limited by short sampling durations (compared to a typical sonications of several seconds or more), and a limited number of investigated input powers, or tip-vibration amplitudes.

More recently, Tan & Yeo [20] investigated a 20 kHz horn with a 12.62 mm-Ø tip, with high-speed imaging of 100, 000 frames per second (fps), over 1.8 s of a 5 s sonication, with simultaneous hydrophone monitoring. Although the main objective of this study was to investigate bubble dynamics within mm-sized channels under the horn, free-field characterisation at tip-vibration amplitudes of 6, 30, 60 and 120 μm was provided. The authors presented spectra < *f_0_* for the acoustic signal collected, which featured subharmonic peaks at *f_0_/m*, with *m* increasing for larger amplitudes, up to *f_0_/8*. The authors speculated that the shift to larger *m* at higher power could be due to a decrease in bubble resonance at higher tip-vibration amplitudes.

Although the literature described reports rather different bubble characteristics observed via direct imaging, the subharmonic collapse behaviour with corresponding pressure spikes in the acoustic data appears to be consistent. In the current paper, a study based on dual-perspective high-speed imaging of cavitation at the tip of a horn and parallel acoustic detection, capturing data for the duration of 2 s sonication, over twenty-five input powers, is reported. We seek a detailed characterisation of the subharmonic response with tip-vibration amplitude (input power), and demonstrate the contribution of periodic shock waves to non-linear features within the noise spectrum of the cavitation emission signal.

## 2 Materials & Methods

### 2.1 The experimental arrangement

The results described below were obtained with a commercial Branson 450 W Digital Sonifier, operating at 20 kHz through a 230 mm long tapered Ti probe, with a ¼” diameter (6.4 mm-Ø) tip. Input power is entered as a percentage value, with a minimum of 10% and programmable in 1% increments. All data was collected from sonications of 2 s duration, initiated manually via a button on the front panel of the control console.

**Fig. 1:**
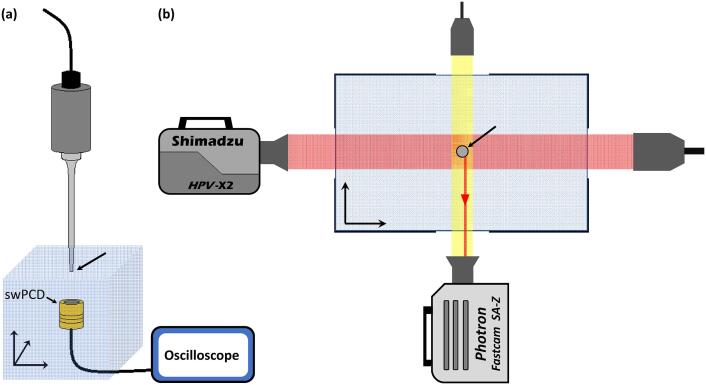
Schematic representations of the experimental arrangement (a) the ultrasonic horn and swPCD. (b) top-down view of the dual-perspective high-speed imaging configuration. The horn-tip is arrowed in both representations.

The horn was mounted such that the tip was submerged 25 ± 1 mm below the surface of de-ionised water, within a tank measuring 420 × 438 × 220 mm^3^, Fig. 1. High-speed imaging, as described in §2.3, was conducted through glass windows embedded within the walls of the tank, Fig. 1(b).

### 2.2 Dual perspective high speed imaging

High-speed imaging of the cavitation at the tip during a sonication was undertaken with two high speed cameras, from orthogonal perspectives in the horizontal plane, Fig. 1(b). A Fastcam SA-Z 2100 K (Photron, Bucks UK) recorded the bubble activity for the entire 2 s duration of each sonication reported at 1 × 10^5^ frames per second (fps), with a 159 ns shutter time, through a macro-lens (EX DG, 24–70 mm 1:2.8, Sigma, Hertfordshire UK) and a 36 mm extension tube. At this frame rate, imaging was achieved over 384 × 256 pixels, with a resolution of 25 μm/pixel. Illumination was provided by a continuous 150 W halogen bulb source (Thorlabs, Ely UK) coupled to a liquid light guide and a collimating lens. The second high-speed camera was a Shimadzu HPV-X2 (Shimadzu, Kyoto Japan), imaging at a higher temporal resolution of 2 × 10^6^ fps (Mfps), but over a shorter duration of 128 µs. A spatial resolution of 65 μm/pixel was achieved over 400 × 250 pixels through a macro-lens (Milvus 100 mm f/2M, Zeiss, Oberkochen, Germany). Illumination was provided via synchronous (to frame capture) 10 ns laser pulses at 640 nm (CAVILUX Smart, Cavitar, Tampere Finland), coupled to a liquid light guide and a collimating lens. This configuration facilitates shadowgraphic imaging such that pressure transients such as bubble-collapse shock waves are directly imaged via refractive index variations imposed as the shock wave propagates [21], [22], [23], [24]. Indeed, the primary purpose of this imaging was to identify a cluster collapse shock wave, and its incidence to the swPCD, as a reference for the analysis of the Photron imaging and acoustic data.

### 2.3 Acoustic detection

Acoustic detection was undertaken with an in-house fabricated passive cavitation detector, designed for high-sensitivity to broadband (bubble-collapse) shock waves [25], mounted 15 ± 1 mm below the horn-tip, on a *xyz* manipulator, Fig. 1 (a). The shock wave passive cavitation detector (swPCD) is constructed from a 15 mm-Ø disk of 110 μm polyvinylidene fluoride (PVdF) film, with a heavy Tungsten-epoxy backing layer to lower resonance. An epoxy matching layer also provides device robustness, with a sensitivity above instrumental noise up to 4 MHz, reducing with increasing frequency. A comprehensive performance evaluation for the detection of bubble-collapse shock waves, including from acoustically driven cavitation, is available [25]. We note that in this experimental configuration the swPCD will have poor temporal resolution for detection of shock wave emissions from tip-cavitation. The location does, however, put the front-face of the swPCD within the FOV of the Shimadzu imaging, such that individual shock-wave incidence can be observed, and used to identify corresponding features in the swPCD data. Preliminary tests with the swPCD ~ 10 cm from the horn-tip indicated that the positioning at 15 ± 1 mm did not influence cavitation dynamics within the vicinity of the tip, and shock wave periodicity, as reported below, was unaffected.

The swPCD was connected to an oscilloscope (Tektronix 5 series, Berkshire UK) and data sampled at 25 × 10^6^ samples/s. A filtering protocol is applied to reduce noise (low-pass < 10 MHz) and *f_0_* (high-pass > 20 kHz), revealing shock wave content for presentation in the voltage–time domain, and in the frequency domain via application of a fast Fourier transform and Blackman window over the time interval stated (MATLAB, MathWorks).

### 2.4 Data collection

Sonications were initiated manually from the control console of the ultrasonic horn, at prescribed input powers. The rest of the instrumentation was synchronised via electronic triggering from a delay generator (DG535, Stanford Research Systems, Sunnyvale USA). The Photron imaging and swPCD were set up to record the entire duration of each sonication, with the Shimadzu and the pulsed laser illumination triggered 1 s after Photron imaging commenced. We note that following initiation of any given sonication, the cavitation beneath the tip undergoes a ‘growth’ phase, as it builds up toward the ‘stable’ size associated with the particular input power. Larger clouds at higher input powers exhibit longer growth phases. The Photron imaging for the data presented below indicates that the bubble-clouds at all powers have reached their stable size by within 1 s of sonication initiation, such that typical horn operation is represented and analysed. Any datasets for which the Photron imaging did not capture the initiation of bubble activity beneath the tip were discarded. We note that at lower input powers <20%, cavitation occurrence was inconsistent. Results below are therefore limited to the range of 20 – 100 %.

## 3 Results

The results sections below are organised as follows: §3.1 details dual-perspective imaging and swPCD data over a short 300 µs duration, from approximately 1 s into a 2 s sonication at an input power of 33%. A dark-pixel counting algorithm (MATLAB, MathWorks), used to quantify the oscillation behaviour of the ‘summed bubble area’ throughout a sequence of images, is introduced. Some care should be taken in interpreting the dark-pixel counting data presented, not least as the cavitation at the tip extends through 3-dimensions. The simplistic approach is, however, sufficient for identifying cloud-ensemble oscillations and collapse times [19], [26].

The purpose of this section is to characterise the various cavitation behaviours in the vicinity of the tip, and justify the approach adopted for analysis of the subsequent *Results* section. §3.2 presents dark-pixel counting from the Photron imaging, with the corresponding swPCD data over representative 2.5 ms durations, at five selected input powers (justified later, §3.4). Here, we seek to highlight differences in cavitation behaviour at the selected input powers. Table 1 extends these findings via analyses of 200 ms sections of swPCD data, from five individual 2 s sonications at each of the five input powers. §3.3 considers the noise spectra of the emission signal collected by the swPCD, for representative sonications at each of the selected input powers, for both the 200 ms section, and the entire signal collected over the 2 s sonication. This section confirms that the differences in the oscillation behaviour of the cavitation identified in §3.2 are also manifested in the acoustic emission signal generated by the cavitation at each input power. Finally, §3.4 presents the average root mean square of the swPCD signals collected over five 2 s sonications, at twenty-five input powers. This graph is interpreted in terms of the findings of the previous sections.

**Table 1:** *mT_0_* periodicity of primary cluster collapse shock waves, as a percentage of the total number of shock waves detected, from the 200 ms sections sampled. Average shock wave amplitude, ${\overset{-}{V}}_{\mathrm{SW}}$, and total duration over which no shock waves were detected, ΣdNSA, are also given, for each of the selected input powers.

**Input Power**	**Tip-vibration amplitude (*µm*)**	***T_0_* (%)**	**2 *T_0_* (%)**	**3 *T_0_* (%)**	**4 *T_0_* (%)**	${\overset{-}{V}}_{SW}$**(*mV*)**	**ΣdNSA (*ms*)**
25%	130 ± 25	1.15 ± 0.43	98.88 ± 0.43	—	—	585.07 ± 5.63	0.96 ± 0.31
33%	155 ± 25	19.08 ± 1.02	35.38 ± 1.88	41.54 ± 1.60	4.00 ± 2.39	680.38 ± 19.77	10.95 ± 0.51
40%	175 ± 25	0.48 ± 0.55	6.02 ± 2.71	93.70 ± 2.79	—	1083.40 ± 22.35	4.28 ± 0.79
50%	219 ± 25	13.30 ± 3.11	30.86 ± 2.30	16.92 ± 3.93	38.92 ± 1.70	1108.20 ± 53.00	13.02 ± 2.21
60%	263 ± 25	0.08 ± 0.11	3.42 ± 2.18	32.92 ± 2.12	62.98 ± 2.79	1400.80 ± 44.07	8.52 ± 2.11

### 3.1 Data registration and cavitation characterisation

**Fig. 2:**
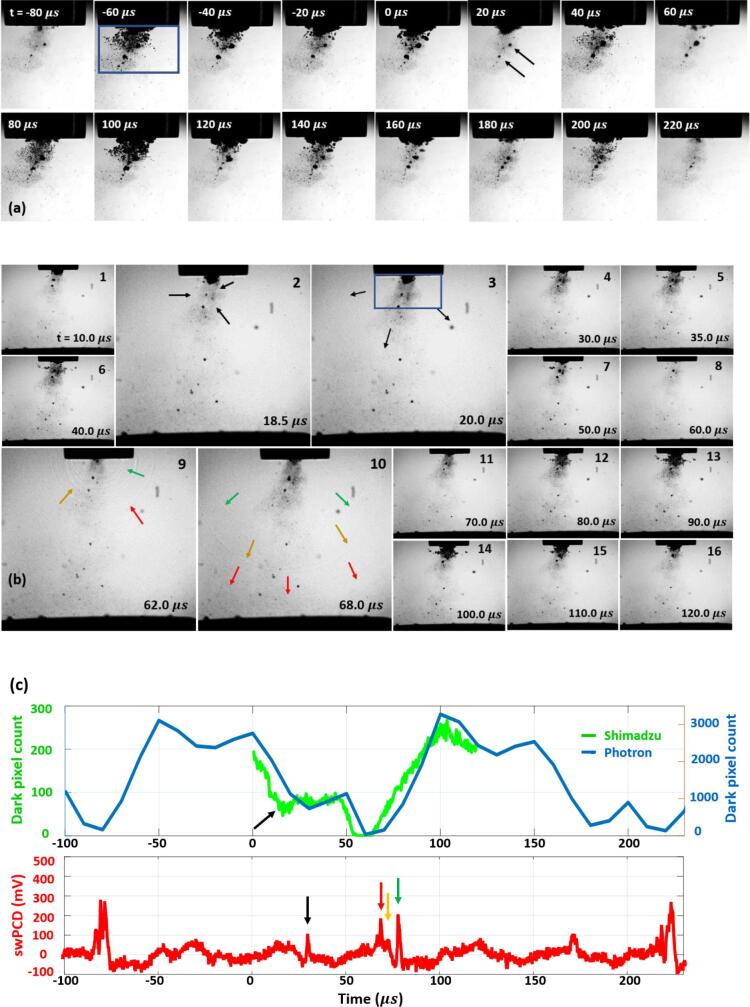
Sample data representing cavitation characterisation and data-registration between: (a) Photron imaging at 1 × 10^5^ fps, (b) shadowgraphic Shimadzu imaging at 2 Mfps. The same two satellite clusters are arrowed in (a) at 20 µs and (b) at 10 µs, with the shock wave generated by the upper cluster also arrowed at 18.5 and 20.0 µs. Coloured arrows at 62.0 and 68.0 µs track shock wave fronts generated by primary sub-cluster collapses, as they propagate through the FOV. Scale for both imaging data perspectives is provided by the 6.4 mm-Ø horn-tip, and t = 0 μs is determined by the start of the Shimadzu imaging. (c) dark pixel counting for both imaging sequences and swPCD measurements.

Fig. 2 presents sample data at a horn input power of 33%, approximately 1 s into a sonication, as collected by (a) the Photron at relatively high spatial resolution, (b) the Shimadzu over a larger field-of-view (FOV) but at high temporal resolution, and (c) the swPCD, the front face of which is visible at the bottom of the Shimadzu images. Image sequences from both perspectives at full FOV are available in movie format, as *Supplemental Materials.*

Both imaging perspectives reveal cavitation activity generally consistent with many previous reports; a primary bubble cluster that remains effectively in contact with the horn-tip throughout, ‘satellite’ clusters that are detached from the tip, and a cloud of smaller bubbles that are not always well resolved, extending to several mm below the tip. The Shimadzu imaging of Fig. 2 (b), further reveals distal clusters > 5 mm from the tip, including along the front face of the swPCD. Over all the observations made in this study, no shock wave was detected from a distal cluster (either via the shadowgraphic imaging perspective or within the swPCD data), confirming they cavitate non-inertially, [27].

The Photron data of Fig. 2 (a) is presented over ~ 300 μs, and thereby captured six full tip-oscillations. The dark-pixel counting algorithm provides an overview of ‘summed bubble area’ behaviour for this duration, Fig. 2 (c, blue), sampled from the region represented by the box below the tip, Fig. 2 (a) at −60 µs. It should be noted that this region includes the primary cluster, satellite clusters and some of the extended small bubble cloud. The primary cluster, however, which represents the largest and most dynamic of the bubble structures will dominate the dark-pixel counting curve. Two peaks in the curve, at −50 and 100 µs, representing frames of maximum inflation as captured by the Photron camera, follow minima in the curve, when the summed bubble area was close to zero at −80 and 60 µs (with a subsequent minima apparent at 220 µs). The frames associated with these summed bubble area minima are presented in Fig. 2 (a), at the respective timings. These images suggest the primary cluster has just collapsed, or is about to collapse, with the exact timing not precisely identified at the 1 × 10^5^ fps temporal resolution of this perspective.

The Shimadzu data of Fig. 2 (b), recorded at 2 Mfps from t = 0 – 128 µs, probes the activity around the Photron dark-pixel counting minimum at t = 60 µs with higher temporal resolution and shadowgraphic imaging, facilitated by the pulsed laser illumination described in §2.3. The illumination for the Shimadzu imaging axis, which is triggered in advance to sequence capture, is faintly apparent within the Photron imaging as some laser light is reflected orthogonally from the horn tip, Fig. 2 (a), and represented schematically, Fig. 1 (b). The two satellite clusters identified in the Photron imaging at t = 20 µs, Fig. 2 (a), are also arrowed in the Shimadzu imaging of Fig. 2 (b), at t = 10.0 µs, for registration between the perspectives. Images 2, 3, 9 and 10 have been enlarged as they feature various cluster collapse shock waves (the propagation of which may be better perceived in the movie version of the data, available as *Supplemental Materials*). The green curve of Fig. 2 (c) represents the output from the dark-pixel counting algorithm applied to the Shimadzu image sequence.

Image 2 of Fig. 2 (b), at 18.5 µs captures a shock wave generated from the collapse of the upper of the two satellite clusters identified, the propagation of which is still faintly apparent at t = 20.0 µs (see *Supplemental Materials* for movie version of this data). The Shimadzu dark-pixel counting curve captures this collapse, with a local minimum of ~ 50 dark pixels arrowed black around 18 µs, and this shock wave is acoustically detected by the swPCD, arrowed Fig. 2 (c), after a propagation time of ~ 10 µs. Image 9 and 10 capture the shock wave fronts generated by the prominent collapse of the primary cluster around 60 µs. The shadowgraphic imaging allows three main shock fronts to be identified along with the corresponding acoustic detections (arrowed red, orange and green, Fig. 2 (b) and (c) respectively). The multiple fronts of this shock wave indicate that the primary cluster has not collapsed uniformly, but as several sub-clusters, each collapsing at slightly different times. This may be verified in the Photron imaging at t = 60 µs, where four sub-clusters are apparent within the region occupied by the primary cluster (arrowed black, Fig. 2 (a)). The µs and sub-µs differences in the collapse timings of these sub-clusters explain why the minimum of the Shimadzu dark-pixel counting curve around this time is somewhat U-shaped. The *Supplemental Material* movie version of this data reveals that as one sub-cluster collapses, the others are either still deflating into collapse or rebounding from it, such that there is not a well-defined minimum. We note that satellite cluster collapses also contribute shock-fronts to the primary cluster collapse shock wave. The extended cloud of smaller bubbles is at its most prominent after excitation via the propagation of a shock wave, from the collapse of either the primary sub-clusters or a satellite cluster. The dark-pixel counting from both imaging perspectives register this excitation as local peaks, with the exciting shock waves either visible through the shadowgraphic imaging, or acoustically detected by the swPCD after propagation delay.

Another feature of Fig. 2 are the partial, non-collapsing deflations captured by the Photron imaging at t = −20, 30, 130 and 180 µs. Taken along with timings of the collapses at t = −80 and 60 µs, primary cluster deflations generally occur approximately at the 50 µs period of the tip-vibration (*T_0_*), with variations in the precise timings attributable to host medium inertia effects retarding the bubble dynamics according to the degree of the cluster deflation/inflation. The swPCD data of Fig. 2 (c) indicates primary cluster collapse, and shock wave generation at 3 *T_0_* (or *f_0_*/3) for the short sampled duration.

To summarise, the cavitation in the region surrounding the tip comprises at least four distinct types of cavitation activity; the primary cluster (and sub-clusters), satellite clusters, an extended cloud of smaller bubbles and distal clusters. The first two are capable of shock wave generation, with the primary cluster generating periodic multi-fronted shock waves at subharmonic values to the tip-vibration, and satellite clusters also generating shock waves independently of the primary cluster oscillations. Both high-speed imaging perspectives, and the parallel acoustic detection of our experimental configuration, are required to characterise and distinguish between the behaviour of these categories of cavitation. The Photron imaging in conjunction with swPCD detection is sufficient, however, to interrogate the subharmonic collapse dynamics of the primary cluster over extended sonication durations, as described in the following section.

### 3.2 Primary cluster shock wave periodicity at the five selected input powers

Having established the utility of the experimental configuration, results are now presented from an investigation to assess the periodicity of primary cluster collapse and shock wave emission, across five selected key input powers of 25, 33, 40, 50 and 60%. The justification for the selection of these particular input powers will be given in §3.4, where data from twenty-five input powers, across the full available range, are considered.

**Fig. 3:**
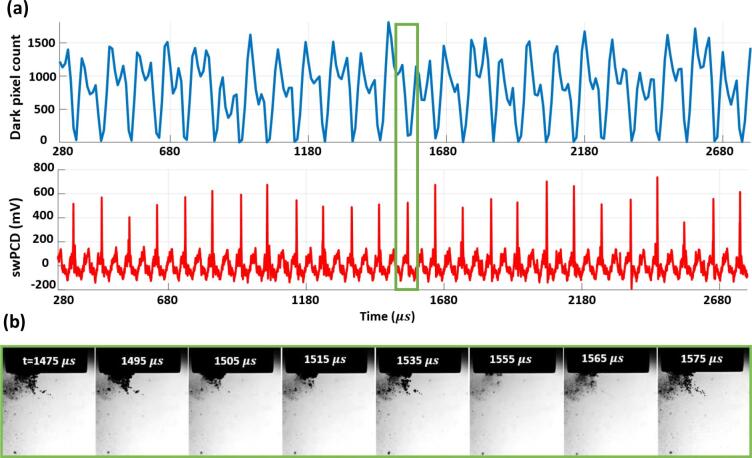
(a) Dark-pixel counting and swPCD data over a ~ 2.5 ms duration from within a 2 s sonication at **25%** input power. (b) Representative Photron imaging from within the duration, corresponding to the green box of (a). The full image sequence is available in movie format as *Supplementary Material*. (For interpretation of the references to colour in this figure legend, the reader is referred to the web version of this article.)

Fig. 3 (a) is the Photron dark-pixel counting and swPCD data, over a 2.5 ms duration approximately halfway through a 2 s sonication, at 25%. Fig. 3 (b) are the source images corresponding to the minimum of the dark-pixel counting curve at 1555 µs, with a corresponding shock wave detected by the swPCD. We note that the cavitation activity is not centrally located with respect to the horn-tip, which was commonly observed for lower input powers.

**Fig. 4:**
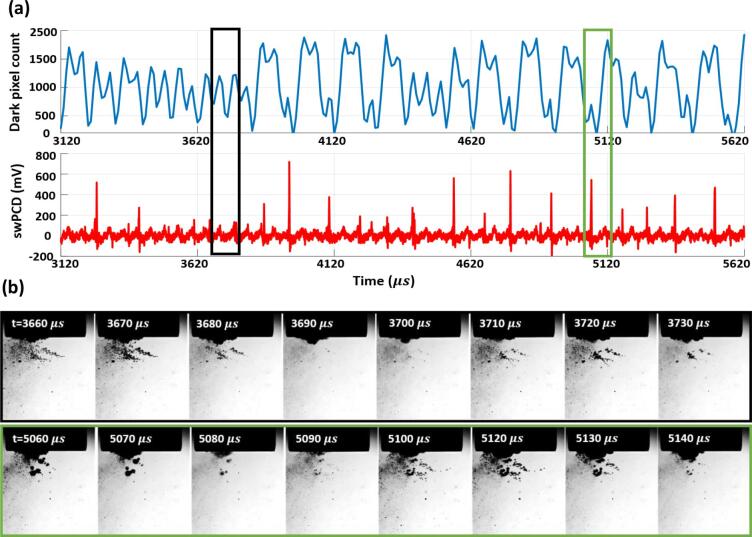
(a) Dark-pixel counting and swPCD data over a ~ 2.5 ms duration from within 2 s sonication at **33%** input power. (b) Representative Photron imaging from within that duration, corresponding to the green and black boxes of (a). The full image sequence is available in movie format as *Supplementary Material*. (For interpretation of the references to colour in this figure legend, the reader is referred to the web version of this article.)

The data of Fig. 3 reveals primary cluster collapse and shock wave detection at regular periodicity of ~ 100 µs, or 2 *T_0_* of the tip-driving. In between each collapse there is typically a single non-collapsing deflation, captured in Fig. 3 (b) around 1515 µs. Fig. 4 presents data at an input power of 33% (as for Fig. 2), but in a format equivalent to Fig. 3, with Fig. 4 (b) displaying the source images over two sections of the 2.5 ms duration, according to the black and green boxes. The frame at 5090 µs was taken around a moment of primary cluster collapse, with the resulting shock wave clearly detected by the swPCD.

The two preceding shock waves detected by the swPCD, with corresponding minima in the dark-pixel counting curve, were emitted at ~ 150 µs intervals, or 3 *T_0_*. The shock waves following the one highlighted, however, are closer to the 2 *T_0_* periodicity that characterised the cavitation behaviour at 25%, Fig. 3.

Earlier in the data represented by Fig. 4, between 3400 and 3800 µs, there appears to be an extended sequence of non-collapsing deflations, with no prominent shock wave typical of a primary cluster collapse, detected by the swPCD.

**Fig. 5:**
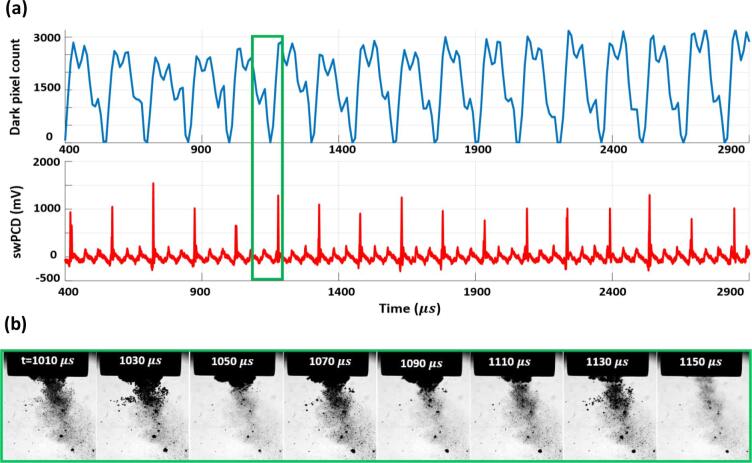
(a) Dark-pixel counting and swPCD data over a ~ 2.5 ms duration from within 2 s sonication at **40%** input power. (b) Representative Photron imaging from within that duration, corresponding to the green box of (a). The full image sequence is available in movie format as *Supplementary Material*. (For interpretation of the references to colour in this figure legend, the reader is referred to the web version of this article.)

At a higher input power of 40%, Fig. 5, a larger cluster exhibits regular 3 *T_0_* collapse behaviour throughout the 2.5 ms duration. The dark-pixel counting curve of Fig. 5 (a) indicates that every collapse, confirmed via the detection of a shock wave in the swPCD trace, is followed by an inflation, a partial deflation, a re-inflation then a stronger but still non-collapsing deflation, before the next successive collapse.

The source images of Fig. 5 (b) indicate that satellite clusters and the extended small bubble cloud will be making contributions to the dark-pixel counting curve, although these will be somewhat limited by the region sampled below the horn, Fig. 2 (a) at −60 µs. The prominent shock waves in the swPCD data of Fig. 5 (b), confirm 3 *T_0_* collapses for the primary cluster.

**Fig. 6:**
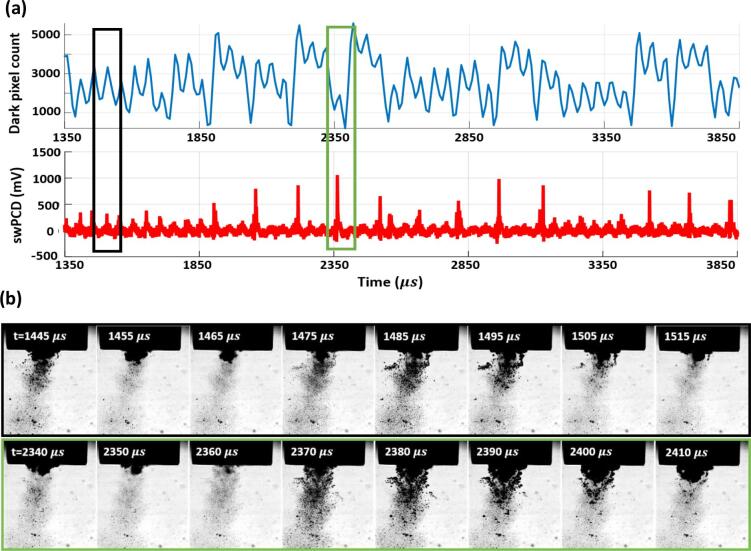
(a) Dark-pixel counting and swPCD data over a ~ 2.5 ms duration from within 2 s sonication at **50%** input power. (b) Representative Photron imaging from within that duration, corresponding to the green and black boxes of (a). The full image sequence is available in movie format as *Supplementary Material*. (For interpretation of the references to colour in this figure legend, the reader is referred to the web version of this article.)

Fig. 6 reveals a mixture of 3 *T_0_* and 4 *T_0_* behaviour at 50%, with the latter behaviour incorporating an additional non-collapsing deflation phase. As for an input power of 33%, Fig. 4, Fig. 6 also exhibits extended durations without any strong collapse shock waves, such as that for which source images are provided within the black box of Fig. 6 (b). The low amplitude (<400 mV) features that are detected by the swPCD, suggest that these shock waves may originate from satellite cluster collapses.

**Fig. 7:**
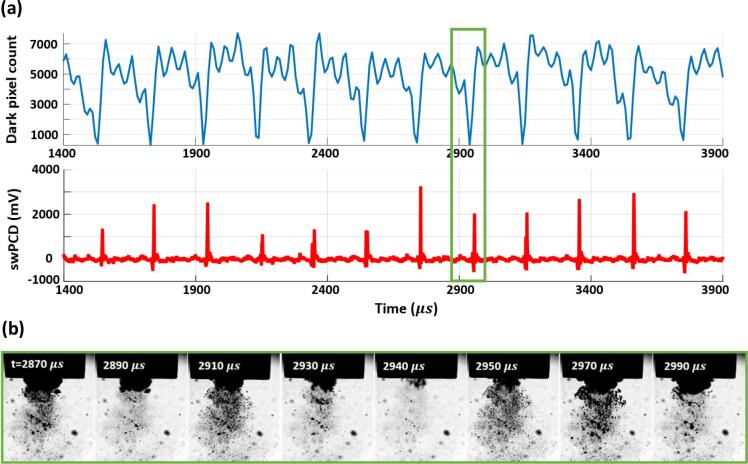
(a) Dark-pixel counting and swPCD data over a ~ 2.5 ms duration of a 2 s sonication at **60%** input power. (b) Representative Photron imaging from within that duration, corresponding to the green box of (a). The full image sequence is available in movie format as *Supplementary Material*. (For interpretation of the references to colour in this figure legend, the reader is referred to the web version of this article.)

Finally, Fig. 7 at an input power of 60%, suggests that regular periodic behaviour has been resumed, but with a periodicity of 4 *T_0_*, such that three non-collapsing deflations now occur between each collapse. As with Fig. 5 at 40%, the final deflation before the collapse of each cycle appears to be the lowest minimum of the non-collapsing deflations.

Fig. 3, Fig. 4, Fig. 5, Fig. 6, Fig. 7 sample raw imaging data obtained for cavitation activity during single sonications at each of the selected input powers. To demonstrate the behaviour identified is representative of the cavitation at these powers, we analysed 200 ms sections of swPCD output, from data collected over five 2 s sonications at these powers, Table 1. Specifically, shock wave periodicity was assessed for the 200 ms section, from t = 0 μs (the start of the Shimadzu imaging) approximately 1 s into each sonication, with the number of shock waves at periodicities of *mT_0_* (for *m*’s between 1 and 4), expressed as a percentage of the total ± the standard deviation across the five data sets collected, at each power. The average value of the shock wave amplitude, ${\overset{-}{V}}_{\mathrm{SW}}$ (mV), across the five sections at each input power, are also given (±the standard deviation). Only shock waves within 5% of the *mT_0_* timings and of amplitude > 20% of${\overset{-}{V}}_{\mathrm{SW}}$, were considered, to discount aperiodic shock waves generated by satellite clusters collapsing in isolation to the primary cluster.

The final column of Table 1 contains the summed durations within which no prominent shock waves were detected by the swPCD, representing the intervals of extended non-collapsing deflations identified in Fig. 4, Fig. 6, over the five 200 ms sections at each power. We refer to this as the *summed duration of non-shocking activity,* ΣdNSA (ms). Table 1 also includes the peak-to-peak tip-vibration amplitudes for the five selected input powers, estimated from the Photron imaging and an assumption of simple harmonic motion for the tip.

To summarise, the dark pixel counting curves of Fig. 3, Fig. 4, Fig. 5, Fig. 6, Fig. 7 (b) indicate that all general oscillations (non-deflating collapses and collapses) occur on a 50 µs timescale, in accordance with *T_0_* of the tip-vibration. Table 1 reveals a trend for *mT_0_*, the time between consecutive collapses to increase through integer values, with *m* = 2, 3 and 4 dominating for input powers of 25, 40 and 60%, respectively. Practically, this manifests as an additional non-collapsing deflation between successive collapse phases, as the power is increased from one of these values of input, to the next. At the intermediate values of 33 and 50%, *m* is less distinct. Notably, at 33% input power, more collapses occur at *T_0_*, than at 25%. Similarly, more collapses occur at 2 *T_0_* for 50%, than at 40%. The ΣdNSAs are also significantly longer at these intervening powers, reflecting the swPCD data presented in Fig. 4, Fig. 6.

### 3.3 Cavitation emission noise spectra at the five selected input powers

In this *Results* section, data from the swPCD at the five input powers are presented in spectral form. Song *et al* [22] provides a model for determining the contribution of periodic bubble-collapse shock waves to the noise spectrum of the emission signal, whereby the frequency of shock wave emission, *f_sw_* generates spectral peaks at *nf_sw_*, for all *n*. For shock waves emitted at subharmonic values of the fundamental driving frequency, *f_0_*, spectral peaks are therefore raised at *nf_0_/m*. Scattering of the primary field contributes further to the *f_0_*-peak, and *nf_0_* peaks in the case of non-linear propagation.

**Fig. 8:**
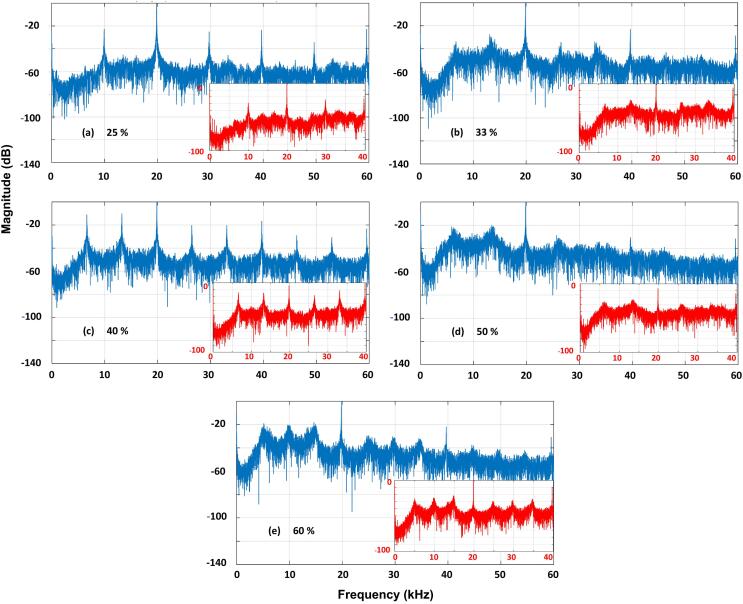
Cavitation emission noise spectra for the sampled 200 ms section of the signal, at the five key tip-vibration amplitudes of (a) 25, (b) 33, (c) 40, (d) 50 and (e) 60%. The insets represent the spectrum for the entire 2 s sonication.

Fig. 8 (a-e) provides representative noise spectra of the signal collected by the swPCD, during a sonication at each of the selected input powers of §3.2, for both the 200 ms duration of signal analysed for Table 1, and the entire 2 s sonication (insets). The similarity between the 200 ms spectrum and the whole-signal spectrum at each power, may be taken to indicate that the 200 ms section is representative of the entire sonication.

At 25% Fig. 8 (a), the lowest of the selected input powers, the spectrum exhibits clear peaks (~20 dB above the local noise floor) with *m* = 2 at *nf_0_/2*, for all *n*. The spectral model of [22] suggests that the 2 *T_0_* periodic shock waves generated by the main cluster collapses, Fig. 3 (a) and Table 1, raise these peaks, with additional contributions to *nf_0_* (with *n* = 2 within the presented bandwidth), arising from the primary field. Equivalently, the 3 *T_0_* shock waves of Fig. 3 (c), at 40% input power, raise the clear *nf_0_/3* peaks of Fig. 8 (c).

At the intermediate power of 33%, the periodicity of shock wave emission is less distinct, and accordingly, the spectral peaks are not so well formed, Fig. 8 (b). The small peaks of several dB at *nf_0_/3*, are attributable to the > 40% shock wave emission at 3 *T_0_*, Table 1. We introduce the term *transitional input power* to describe tip-vibrations that generate main-cluster oscillations *transitioning* between subharmonic orders of *m*.

This trend repeats for the higher input powers, with *m* transitioning between 3 and 4 at an input power of 50%, and *m* = 4 emerging at 60%, for > 60% periodicity at 4 *T_0_*, Table 1. It should be noted that at the higher input powers, any given shock wave periodicity appears to be less dominant, even at non-transitioning tip-vibration amplitudes.

**Fig. 9:**
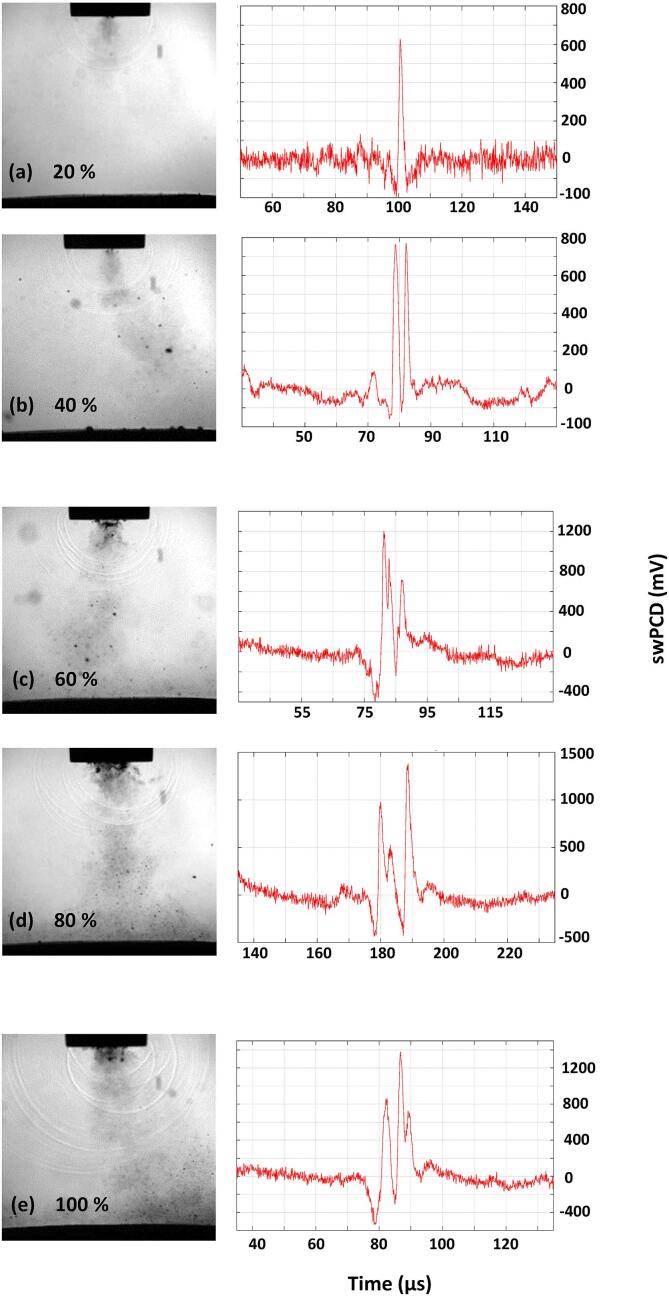
Single Shimadzu frames capturing a shock wave generated by a primary cluster collapse at increasing input powers of (a) 20, (b) 40, (c) 60, (d) 80 and (e) 100%, representing the tendency for higher numbers of component shock-fronts at higher powers. Full image sequences are available in movie format as *Supplementary Materials*.

Song *et al* [24] extended the spectral model of periodic shock waves, [22] to demonstrate experimentally that for contrast agent microbubbles driven to cavitate by focused ultrasound, the spectral floor (over instrumental noise) is determined by variations in the amplitude of the shock waves generated, and the precise timings of shock wave emission. These variations redistribute power that would otherwise be contained within the frequency peaks, to broadband components. Fig. 3, Fig. 4, Fig. 5, Fig. 6, Fig. 7 and Table 1 of §3.2, reveal the amplitude variance in the detected shock waves is disproportionately higher at the transitional input powers, than those of the non-transitional powers. Moreover, Fig. 9 (a-e) represents the general trend for primary cluster collapse shock waves at higher input powers, to consist of a higher number of component shock-fronts, across the full range of powers available (20–100%). Full image sequences from which the frame presented in Fig. 9 was extracted, are available in movie format, as *Supplemental Materials*. As described previously, §3.1, each shock-front is generated by the individual collapse of a sub-cluster within the primary cluster. Larger primary clusters contain more sub-clusters, generating more component shock-fronts on collapse, with additional contributions from an increased number of satellite clusters, at higher powers.

Note that not all shock wave fronts apparent in the high-speed image of Fig. 9, are resolved by the swPCD in this position, as described in *§2.4 Acoustic detection*. For the noise spectra of the acoustic emission signals, Fig. 8 (a-e), the increased variance in shock wave amplitudes at the transitional input powers, and multi-fronted shock waves generated at higher input powers, will both contribute to raising the noise floor of the spectrum. These effects are perceptible above the lower sensitivity threshold for swPCD, apparent at 3–4 kHz.

### 3.4 Shock wave content within the emission signal over twenty-five input powers

**Fig. 10:**
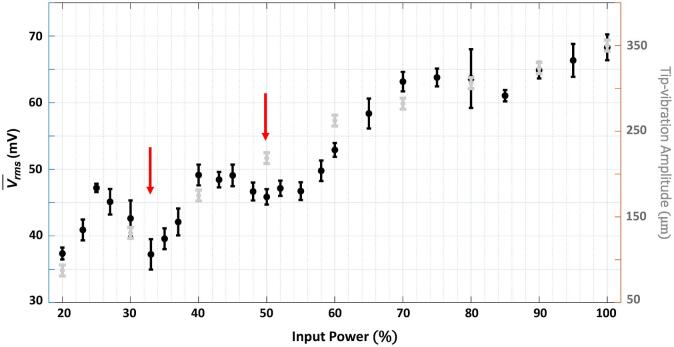
The mean-*V_rms_*, from five 2 s sonications at each input power, of the signal collected by the swPCD, over twenty five input powers. Error bars represent the standard deviation within the five data sets. The peak-to-peak tip-vibration amplitude, estimated from the Photron imaging data is also presented, ±25 µm (pixel resolution for this imaging perspective).

In this final result section, swPCD data at twenty-five input powers between 20 and 100% is presented. The cavitation emission signals collected for five 2 s sonications, at input powers in 5% increments, were initially considered. The root mean square of the voltage, $V_{\mathrm{rms}}$ , for the signal collected from each sonication, is taken to quantify the time-averaged shock wave content within the signal. Fig. 10 represents the mean-$V_{\mathrm{rms}}$, ($\overset{-}{V}$*_rms_*), over the five sonications, with error bars representing the standard deviation. Extra data were subsequently collected around ‘input powers of interest’, in terms of the overall structure of the plot*.*

To remove the possibility that any interpretation of Fig. 10 may be due to non-linear response of the acoustic element driving the tip-vibration, or amplification of the vibration through the tapered rod of the horn, values for the tip-displacement amplitude at 10% input power increments are provided (grey data, Fig. 10). These were estimated from the Photron imaging, with an accuracy primarily limited by the pixel-resolution of this imaging perspective.

Fig. 10 reveals local mimina (arrowed red) in $\overset{-}{V}$*_rms_*, at the transitional input powers of 33 and 50%, corresponding to the indistinct values of *m*, or orders of subharmonic collapse, consistent with those identified in §3.3 and 3.4. The plot also indicates another transitional input power at ~ 85%, possibly due to *m* transitioning from 4 to 5, although the data is much noisier at higher amplitudes. We attribute the minima to extended periods of non-collapsing deflations, ΣdNSA Table 1, in comparison to the regular, concerted periodic shock waves generated by primary cluster collapses, at the input powers with distinct *m*.

## 4 Discussion

The results presented indicate that oscillations of the primary cluster at the tip of the ultrasonic horn progresses through orders of subharmonic response, with *m* increasing through integer values as amplitude of tip-vibration (input power) increases. In practice, this means the cluster undergoes *m* – 1 non-collapsing deflations, between each collapse cycle. The 2 *T_0_* subharmonic behaviour of the primary cluster at an input power of 25% (peak-to-peak tip-vibration amplitude of 130 ± 25 µm), Fig. 3 (a), is generally known as *period-doubling* [28]. This effect is attributed to the inertia of the host liquid preventing bubble structures that inflate to a sufficient size during a tension phase, undergoing full deflation-to-collapse with every compressive phase of the driving. The progression through the integer values of *m* exhibited by the primary cluster, is analogous to that reported for a single cavitation cluster driven by 220 kHz focused ultrasound, at increasing values of pressure amplitude [21]. Regimes of indistinct *m* at intervening values of pressure amplitude, were also reported.

In terms of subharmonic components in the spectrum of the acoustic emission signal, the progression with increased driving has been recognised for some time [29], [30]. The dual perspective high-speed imaging and acoustic detection configuration described here, in conjunction with the spectral model for periodic shock waves [22], [24], confirms that the subharmonic emission components are mediated via the shock waves generated by the primary cluster collapses, at increasing *m* for higher input powers, §3.3. The shadowgraphic imaging component at high temporal resolution was required to characterise the cavitation in the general vicinity of the tip §3.1, particularly to distinguish the shock waves generated by the primary cluster from those emitted by proximal satellite clusters.

As reviewed in the *Introduction*, the cavitation at the tip of an ultrasonic horn has received significant research attention. Although many studies have investigated the cavitation at different tip-vibration amplitudes, most are limited to a small number of amplitudes or power values. We believe Fig. 10, over twenty-five input power values, is the most comprehensive investigation over the range of powers available for a given horn configuration, to date. Crucially, this parameter space is sampled sufficiently to reveal the transitional input powers as *m* switches from 2 to 3 at 33% (155 ± 25 µm), and 3 to 4 at 50% (219 ± 25 µm), in terms of time-averaged shock wave content in the emission signal, $\overset{-}{V}$*_rms_*. The transitioning can then linked to the specific cavitation behaviour observed over shorter durations within each sonication, Fig. 4, Fig. 6 of §3.2, and the general shock wave characteristics of Table 1.

Žnidarčič *et al* [19] previously reported on high-speed observations and acoustic detection of tip-cavitation, at a single value of input power, that is comparable to what we have observed at the transitional input powers. The authors describe ‘irregular cavitation dynamics’ with an ‘inability to lock to either 3 or 4 acoustic [tip-vibration] cycles’, with the acoustic data revealing durations of reduced amplitude shock wave emissions, of no discernible periodicity. Our results indicate that this study identified transitional cavitation behaviour at just one of the input powers for which it will occur.

A number of other studies have presented acoustic emission data in terms of a measurement representing the intensity or energy detected during a sonication, as a function of input power or tip-vibration amplitude. Tzanakis *et al.* [31], reporting on the use of a novel high-temperature ‘cavitometer’ for measuring cavitation in molten metal, presented *V_rms_* from the detection of sonications generated by a 20 kHz sonotrode, with a 15 mm-Ø tip in water. Data was provided for tip-vibration amplitudes from up to 45 µm in ~ 5 µm increments, analogous to Fig. 10 above, which featured a local minimum at an amplitude of 18 µm. Hodnett *et al.* [32], using the same 450 W Branson horn that we have used for this study, *Materials & Methods* §2.1, but with a 12.7 mm-Ø tip, measured sonications with a cavitation sensor developed at the National Physical Laboratory (NPL). This sensor, [33], [34] is constructed from 110 µm PVdF film, which also provides the active element of the swPCD, but in a cylindrical geometry such that the tip of the horn can be located centrally within the device. Broadband energy from the spectra of sonications were presented as a function of ‘output setting’ (equivalent to input power, for this paper) in 10% increments, with a local minimum detected at 80%. Both reports suggest the reduction in intensity or energy may be due to cavitation shielding, whereby bubbles located between the source cavitation intended for detection, and the detector, scatter and attenuate the source cavitation emissions, at the particular power/tip-vibration amplitude in question. The results described from the current work suggest that these measurements may have been taken at transitional amplitudes, as an alternative explanation. Indeed, we did not observe any perceptible cavitation shielding effects influencing the shock waves generated by periodic primary cluster collapses. We further note that full identification of all the transitional input powers associated with any particular ultrasonic horn configuration would require sampling at sufficiently fine increments of tip-vibration amplitude, across the full range of input powers available.

For the diverse range of ultrasonic horn applications, and of acoustic cavitation generally, sonications at transitional input powers may be expected to generate seemingly spurious results in terms of application ‘yield’. Fig. 10 indicates that increasing power to the piezoelectric transducer does not necessarily translate to an increased intensity of cavitation activity, as determined by the level of detectable shock wave content within the emission signal. This may be particularly the case for applications such as sonochemistry, known to be mediated by the energetic conditions generated during cavitation collapse [35].

## 5 Conclusion

The cavitation generated by an ultrasonic horn is investigated as a function of input power, with dual-perspective high-speed imaging and detection of the acoustic emissions. The primary cavitation cluster directly underneath the tip collapses subharmonically at *f_0_/m,* with *m* increasing through integer values for increasing tip amplitude. Transitional input powers, where *m* is not well defined, are identified via the *V_rms_* of the acoustically detected signal and its spectrum.

## CRediT authorship contribution statement

**Lukman Yusuf:** Methodology, Software, Visualization, Data curation, Formal analysis. **Mark D. Symes:** Resources, Funding acquisition, Writing - review & editing. **Paul Prentice:** Conceptualization, Methodology, Writing - original draft, Supervision, Funding acquisition.

## Declaration of Competing Interest

The authors declare that they have no known competing financial interests or personal relationships that could have appeared to influence the work reported in this paper.
